# Transcatheter Aortic Valve Replacement for Aortic Regurgitation in Patients with Left Ventricular Assist Devices: An Institutional Experience

**DOI:** 10.1016/j.jscai.2025.103662

**Published:** 2025-05-02

**Authors:** Hanad Bashir, Gustavo Mendez-Hirata, Christian W. Schmidt, Alan Wong, Janelle Muuse, Gregory F. Egnaczyk, Dean J. Kereiakes, Puvi Seshiah, Raviteja R. Guddeti, Nadia El-Hangouche, Santiago Garcia

**Affiliations:** The Christ Hospital Heart and Vascular Institute and the Lindner Center for Research and Education, Cincinnati, Ohio

**Keywords:** aortic regurgitation, left ventricular assist device, transcatheter aortic valve replacement

## Abstract

**Background:**

Aortic regurgitation (AR) is a common complication in patients with left ventricular assist devices (LVAD). However, there is paucity of data regarding the feasibility and safety of transcatheter aortic valve replacement (TAVR) in this population. Hence, we sought to describe the clinical characteristics and outcomes of patients with LVAD and AR who underwent treatment with TAVR at our institution.

**Methods:**

We included all patients with a LVAD who developed clinically significant AR and received TAVR at The Christ Hospital in Cincinnati, Ohio. Baseline clinical and echocardiographic characteristics were collected, and outcomes were defined using Valve Academic Research Consortium 3 criteria.

**Results:**

A total of 7 patients with LVAD were included. The median time from LVAD implantation to TAVR was 3.67 years (IQR, 1.96-4.26 years). The mean age of the patients was 68.6 ± 13.7 years, and the average Society of Thoracic Surgeons score was 6.2 ± 5.2. All patients presented with moderate to severe AR and New York Heart Association functional class III or IV symptoms. All procedures were performed via transfemoral access, with a median procedure time of 149 minutes (IQR, 146-150 minutes). The transcatheter heart valves implanted included commercially available devices—Abbott Navitor (n = 2) and Medtronic Evolut (n = 3)—and dedicated investigational devices for AR—JenaValve Trilogy (n = 1) and J-Valve (n = 1). All patients were discharged alive with no or mild residual AR. There was 1 case of device embolization, which was treated with a second valve, and 1 valve migration treated with snaring and repositioning of the valve. Both complications occurred in patients treated with commercially available self-expanding valves.

**Conclusions:**

TAVR for AR in selected patients with LVAD is a feasible therapeutic option that may improve outcomes. Larger studies are necessary to better define procedural risks, optimal patient selection, and the role of TAVR valves specifically designed for AR in this population.

## Introduction

Aortic regurgitation (AR) is a common complication in patients with durable continuous flow (CF) left ventricular assist devices (LVAD). It has been shown that up to 25% of patients with HeartMate II and HeartWare HVAD durable LVAD develop clinically significant AR within 1 year of implantation.^1^, ^2^, ^3^ Continuous high-velocity flow through the outflow graft creates stress on the aortic valve components, leading to leaflet deterioration, commissural fusion, sinus dilation, reduced smooth muscle cell density, and proximal aorta dilation.^4^, ^5^, ^6^, ^7^, ^8^, ^9^ In addition, microthrombi can develop on the valves and trigger inflammation and degradation.^10^^,^^11^

Aortic regurgitation in patients with LVAD has been reported to decrease forward output, which elevates left ventricular filling pressures leading to recurrence of heart failure symptoms. Chronic significant AR can eventually lead to postcapillary pulmonary hypertension, septal displacement, right heart failure, cardiorenal syndrome, and dysrhythmias.^12^^,^^13^ In patients with LVAD, the development of AR has been associated with a higher risk of death, hospital readmission,^14^ and worsening of right heart failure.^15^

No transcatheter heart valve (THV) has received regulatory approval to treat AR in the United States. Therefore, off-label use of commercial THV devices designed for aortic stenosis has been described in clinical practice.^16^ However, unlike patients with aortic stenosis, patients with AR lack significant annular calcium for adequate anchoring of THV, leading to an increased risk of paravalvular leak (PVL), valve migration or embolization, and need for a second THV, altogether leading to worse patient outcomes. There is paucity of data that describes the safety, efficacy, and outcomes of TAVR in patients with CF LVAD.^17^, ^18^, ^19^ Hence, we sought to describe from an institutional experience the frequency and clinical outcomes of patients on CF LVAD who underwent TAVR for treatment of AR.

## Methods

### Study design

We conducted an observational study that included all patients with LVAD who underwent TAVR for clinically significant AR between 2014 and 2024 at a single institution (The Christ Hospital Health Network, Cincinnati, Ohio). Findings were reported following the recommendations of the Strengthening the Reporting of Observational Studies in Epidemiology (STROBE) statement. One patient with CF LVAD underwent TAVR for aortic stenosis and was excluded from the analysis. We collected baseline clinical, imaging, procedural characteristics, and follow-up data using standardized data collection forms modeled after the TVT registry.^20^ Clinical end points were defined according to the Valve Academic Research Consortium 3 definitions.^21^ Outcomes were defined as intraprocedural and postprocedural complications, valvular echocardiographic parameters, ventricular echocardiographic parameters, and survival at discharge and at 30 days.

### Statistical analysis

Continuous variables are presented as mean ± SD if normally distributed or median (IQR) if skewed. Discrete variables are presented as counts and percentages. All analyses were performed using Stata version 17.0 (StataCorp LLC). The study was approved by the local institutional review board. Informed consent was waived.

## Results

From January 2014 to October 2024, 348 LVAD were implanted in our institution. Of those 348, 7 (2%) patients received a TAVR for significant AR and were included: 1 with a HeartWare HVAD (Medtronic), 1 with a HeartMate II (Abbott), and 5 with HeartMate III (Abbott). Baseline, preprocedural echocardiogram, and right heart catheterization characteristics are presented in Table 1. The mean age of the patients was 68.6 ± 13.7 years, and the average Society of Thoracic Surgeons score was 6.2 ± 5.2. All patients presented with New York Heart Association functional class III or IV heart failure symptoms. The mean (±SD) pulmonary artery pressure and pulmonary capillary wedge pressure were 23.0 ± 9.0 and 13.8 ± 9.2 mm Hg, respectively. The mean left ventricular end systolic and diastolic dimensions were 5.07 ± 1.24 and 6.13 ± 1.14 mm, respectively. Detailed preprocedural computed tomography angiography (CTA) characteristics is described in Table 2. All patients had greater than moderate-severe AR.

**Table 1:** Patient characteristics at presentation.

Characteristic	Patient						
	1	2	3	4	5	6	7
Sex	Male	Female	Male	Male	Male	Male	Female
LVAD type	HeartWare HVAD	HeartMate 3	HeartMate 3	HeartMate 3	HeartMate 3	HeartMate 3	HeartMate II
Age at procedure, y	67	70	61	43	74	79	85
STS PROM	2.6	5.11	2.38	1.97	16.7	8.48	6.15
KCCQ-OS	19.79	NA	18.75	36.46	31.77	NA	26.04
NYHA class	IV	III	III	III	IV	III	III
Right heart catheterization	No	Yes	Yes	Yes	Yes	Yes	Yes
RA, mm Hg		9	7	9	16	4	4
RV systolic, mm Hg		32	22	49	32	19	27
mPAP, mm Hg		24	18	31	22	13	16
PCWP, mm Hg		12	11	23	18	4	6
LVEF, %	10	13	20	17	23	17	23
LVESD, cm		5.76	6.1		4.1	6	3.4
LVESD/BSA		3.8	2.8		2.2	2.9	2
FS, %		9	7		11	6	15
IVS, cm		0.86	0.9		0.85	1.1	0.7
LVEDD, cm	7.3	6.3	6.6	7.1	5.3	6.3	4
LVEDD/BSA	3.882979	3.772455	2.882096	3.796791	2.560386	2.985782	2.312139
MR severity	Moderate	Mild	Mild	Mild-Moderate	Moderate	Severe	Mild
TR severity	Mild-Moderate	Moderate	None	Mild	Moderate	Severe	Severe
AR severity	Moderate-Severe	Severe	Moderate-Severe	Severe	Severe	Severe	Moderate-Severe
AS severity	None	None	None	None	None	None	None
AV calcification severity	Minimal	None	Minimal	None	None	Minimal	None
AV annulus area, mm^2^	681.0	498.0	548.0	622.0	471.0	679.3	467.0
AV annulus perimeter, mm	100.0	84.0	83.6.0	90.0	80.0	93.8	79.0

AS, aortic stenosis; AR, aortic regurgitation; AV, aortic valve; BSA, body surface area; FS, fractional shortening; IVS, interventricular septum; KCCQ-OS, Kansas City cardiomyopathy questionnaire; LVAD, left ventricular assist device; LVEDD, left ventricular end diastolic dimension; LVEF, left ventricular ejection fraction; LVESD, left ventricular end systolic dimension; mPAP, mean pulmonary artery pressure; MR, mitral regurgitation; NYHA, New York Heart Association; PCWP, pulmonary capillary wedge pressure; RA, right atrium; STS PROM, Society of Thoracic Surgeons; TR, tricuspid regurgitation.

**Table 2:** Patient aorta measurements as assessed using computed tomography.

Measurement	Patient						
	1	2	3	4	5	6	7
Aortic annulus eccentricity index	0.31	0.24	0.22	0.19	0.29	0.22	0.25
(+4) perimeter	90.6	77.4	88.2	82.7	79.6	95.2	81.9
Aorta annulus perimeter, mm	95.5	72.1	80.8	87.7	80.8	93.8	78.5
Annulus oversize, %	10.5	26.1	26.5	—	—	13.7	16
(−1) Perimeter	100.9	73.3	81.1	91.1	83.7	94.3	81.2
(−2) Perimeter	102.8	76.1	82.9	93.2	83.2	94.9	81.6
LVOT perimeter, mm	107.7	80.3	85.8	96.7	82.7	96.8	82.7
(+4) Area	652.7	476.3	619.1	543.8	504.5	721.7	534.3
Aorta annulus area, mm^2^	680.9	400.3	500.8	594.8	492.8	682.6	471.1
(−1) area	776.4	411.3	509.4	634.1	532.7	685.2	494.2
(−2) area	783.7	438.8	534.3	659.7	530.7	690.2	506.7
LVOT area, mm^2^	868	486.5	566.7	713.6	503	711.7	516.5
Aortic annulus eccentricity index	0.31	0.24	0.43	0.19	0.29	0.22	0.25
LVOT diameter, mm	33.8	25.1	27.5	30.9	25.9	30.4	25.6
(−1) average	31.8	23	26	29.4	25.9	29.9	25.6
(−2) average	32	23.3	26.7	29.6	26.4	29.9	25.3
(+4) average	28.8	24.6	28.1	26.3	25.3	30.3	26.1
Ascending aorta, mm	33.4	37.4	34.0	29.1	31.2	30.7	34.4
Aorta oversizing	12.1	−10.0	29.0	—	—	19.0	25.0
STJ average, mm	30.4	32.2	32.9	26.9	28.4	29.9	31.6
RCA height, mm	20.6	18.6	19.0	16.0	22.6	25.0	16.4
LCA height, mm	15.8	9.2	16.6	16.6	15.4	12.4	9.3
Aorta valve calcification	None	None	None	None	None	Mild	None
Horizontal aorta angle, °	48.0	60.0	43.0	50.0	42.0	50.0	48.0
Outflow cannula length, mm	49.0	50.0	55.3	38.2	54.7	54.8	60.6
HU	154	243	459	342	370	412	568

HU, Hounsfield units; LCA, left coronary artery; LVOT, left ventricular outflow tract; RCA, right coronary artery; STJ, sinotubular junction.

### Procedure characteristics

The median time from LVAD implantation to TAVR was 3.67 years (IQR, 1.96-4.26 years). All procedures were performed via transfemoral access, with a median procedure time of 149 minutes (IQR, 130-152 minutes). In all cases, the LVAD speed temporarily reduced during valve deployment and subsequently returned to baseline following the procedure. This adjustment was made to minimize the risk of device migration during deployment; LVAD flow velocities and heart rate manipulations can be seen in Table 3. The THVs implanted included Abbott Navitor (n = 2) and Medtronic Evolut (n = 3). Additionally, 2 procedures used dedicated investigational devices for AR: 1 JenaValve Trilogy and 1 J-Valve (JC Medical). All patients were discharged alive with no or mild residual AR. Procedure characteristics are shown in Table 4.

**Table 3:** LVAD flow and heart rate variations pre-, during and post-procedure.

Characteristic	Patient						
	1	2	3	4	5	6	7
LVAD speed pre procedure rpm (heart rate)	3100 (65)	5600 (120)	5600 (95)	5800 (110)	5500 (120)	5800 (123)	9600 (110)
LVAD speed during deployment rpm (heart rate)	2700 (79)	5100 (116)	4800 (75)	5200 (100)	4000 (100)	4700 (80)	8000 (100)
LVAD speed post procedure rpm (heart rate)	3100 (72)	5600 (116)	5600 (69)	5600 (84)	5500 (80)	5400 (102)	9200 (90)

LVAD, left ventricular assist device.

**Table 4:** Valve characteristics and patient characteristics at discharge.

Characteristic	Patient						
	1	2	3	4	5	6	7
Days between LVAD and TAVR	717	234	939	1343	1442	1554	4608
Valve sheath access site	Femoral	Femoral	Femoral	Femoral	Femoral	Femoral	Femoral
Valve model	Medtronic Evolut R	Medtronic Evolut PRO+	Abbott Navitor	J-Valve	JenaValve	Medtronic Evolut FX	Abbott Navitor
Valve size, mm	34	29	35	31	27	34	29
Procedure time, min	114	282	130	150	146	152	149
Fluoroscopy time, min	26.5	65.9	48	14	23.6	28.8	23.6
Contrast volume, mL	85.0	150.0	260.0	189.0	120.0	220.0	163.0
Length of stay, d	31	23	4	2	8	4	27
BAV	No	Postdeployment	No	No	No	No	No
Intraprocedural complications	Yes^a^	Yes^b^	None	None	None	None	None
Discharge MR severity	Mild-moderate	None	None	Trace/trivial	None	Moderate-severe	Mild
Discharge TR severity	Mild-moderate	None	None	None	None	None	Severe
Discharge AR severity	Mild	None	None	None	None	None	None
Discharge PVL severity	None	None	None	None	None	Mild	Trace/trivial
Discharge AS severity	None	None	None	None	None	None	None
Discharge status	Alive	Alive	Alive	Alive	Alive	Alive	Alive
Postprocedural complications	None	None	None	None	None	None	Retroperitoneal hematoma
KCCQ 30 d	58.3	N/A	N/A	78.1	51.6	14.6	N/A

AI, aortic Insufficiency; AR, aortic regurgitation; AS, aortic stenosis; BAV, bicuspid aortic valve; LV, left ventricle; LVAD, left ventricular assist device; MR, mitral regurgitation; PVL; paravalvular leak; TAVR, transcatheter aortic valve replacement; THV, transcatheter aortic valve; TR, tricuspid regurgitation.

a Valve migration into the LV with severe AI and hemodynamic compromise requiring snaring and repositioning at the aortic annulus.

b First THV embolization after low implant and migration into LV and snaring requiring deployment of a second THV.

### Procedural complications

We present a detailed description of the 2 intraprocedural deployment and anchoring-related complications encountered.

#### Patient 1: Left ventricular migration of a 34.0-mm Medtronic Evolut R valve

**Figure 1:**
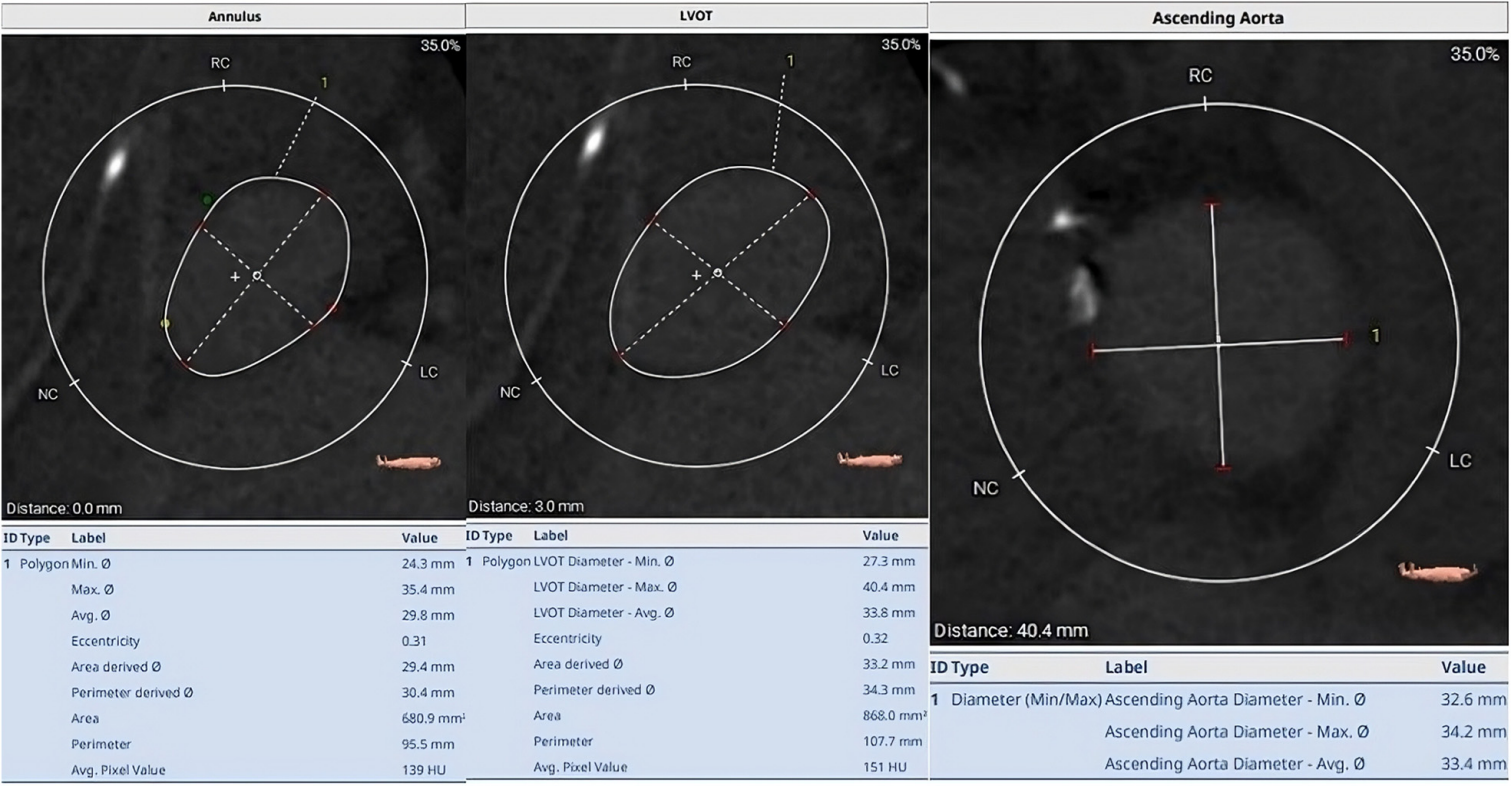
**Patient 1: Preprocedural computed tomography.** LVOT, left ventricular outflow tract.

**Figure 2:**
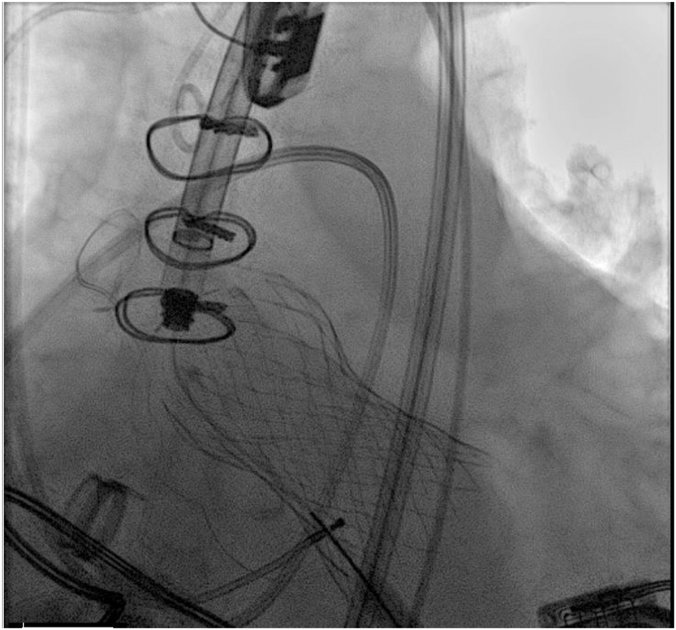
**Patient 1: Migrated 34.0-mm Medtronic Evolut valve.**

**Figure 3:**
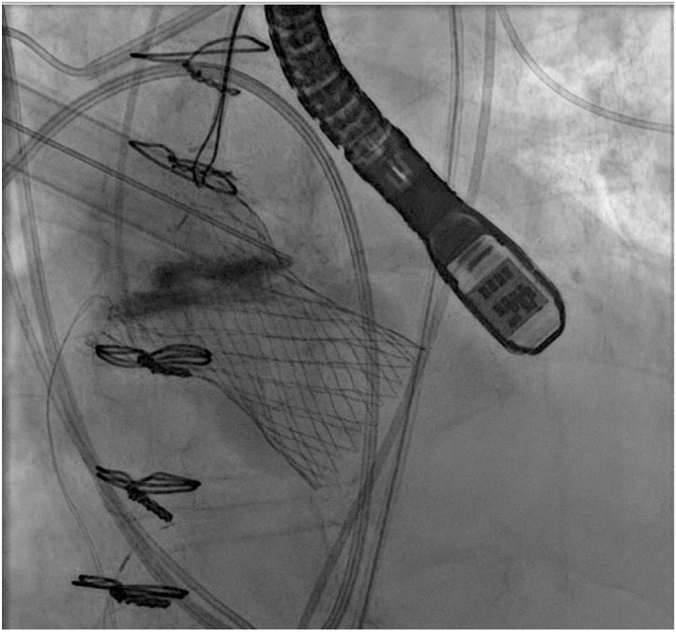
**Patient 1: Repositioned 34.0-mm Medtronic Evolut valve with gooseneck catheter.**

Preprocedural cardiac CTA revealed an annular perimeter of 95.5 mm, an area of 680.9 mm^2^, a left ventricular outflow tract perimeter of 107.7 mm, and an annulus eccentricity index of 0.31 (Figure 1). The calcium aortic score was 0 Agatston units. The valve was deployed 2.0 mm below the noncoronary cusp. Before release, transesophageal echocardiography confirmed adequate valve position and function with trace PVL. The valve was then released slowly but migrated into the LV, leading to wide-open AR and immediate hemodynamic compromise (Figure 2). A 30.0-mm gooseneck snare system was advanced through the guide catheter and used to snare 1 of the THV tabs. It was then carefully maneuvered to lift the THV valve and position the distal portion over the aortic annulus, achieving a good seal with mild PVL. The snare position was maintained for 15 minutes, and aortography revealed a stable, well-seated position 2.0 to 3.0 mm below the annulus (Figure 3). Interrogation of the valve with transesophageal echocardiography showed a well-expanded valve with mild PVL. Retrospective CTA analysis revealed oversizing at the annulus was insufficient at 10.5%, with limited oversizing at the ascending aorta (12.1%) and no oversizing at the level of the left ventricular outflow tract, which had an average diameter of 33.4 mm.

#### Patient 2: Valve embolization of a 29.0-mm Medtronic Evolut PRO+ valve

**Figure 4:**
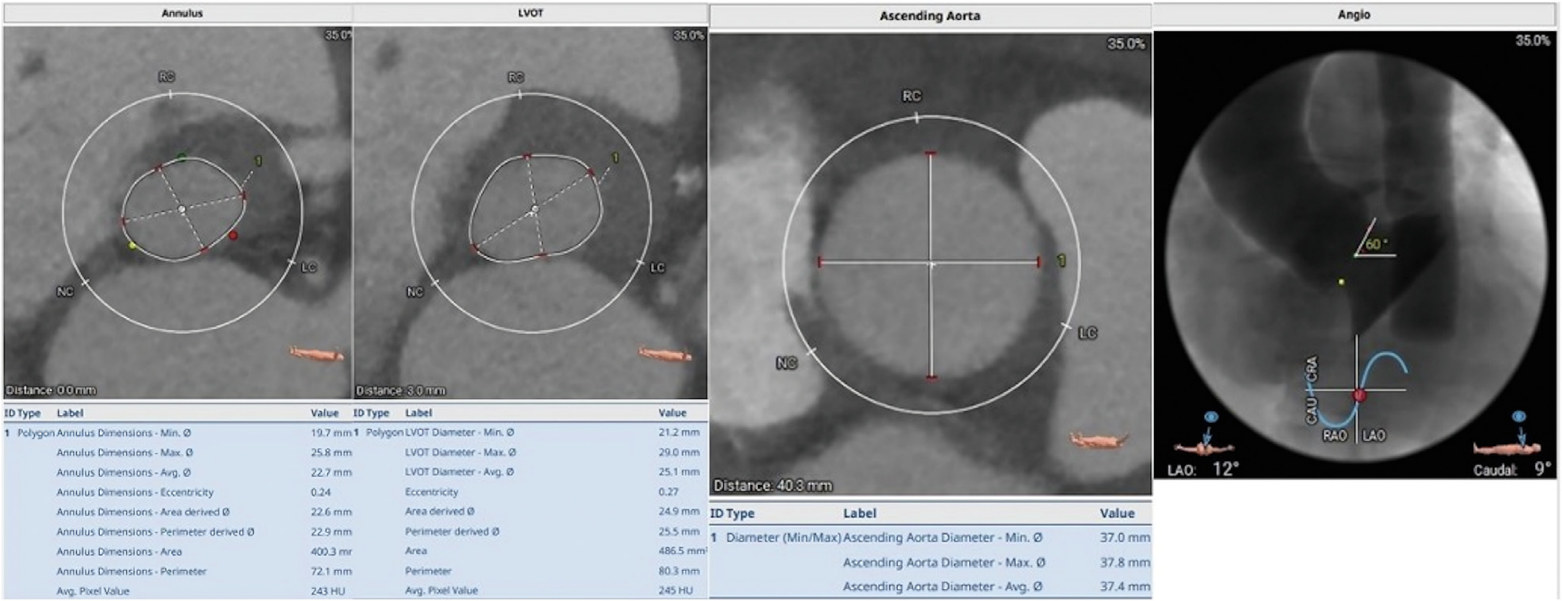
**Patient 2: Preprocedural computed tomography.** LVOT, left ventricular outflow tract.

**Figure 5:**
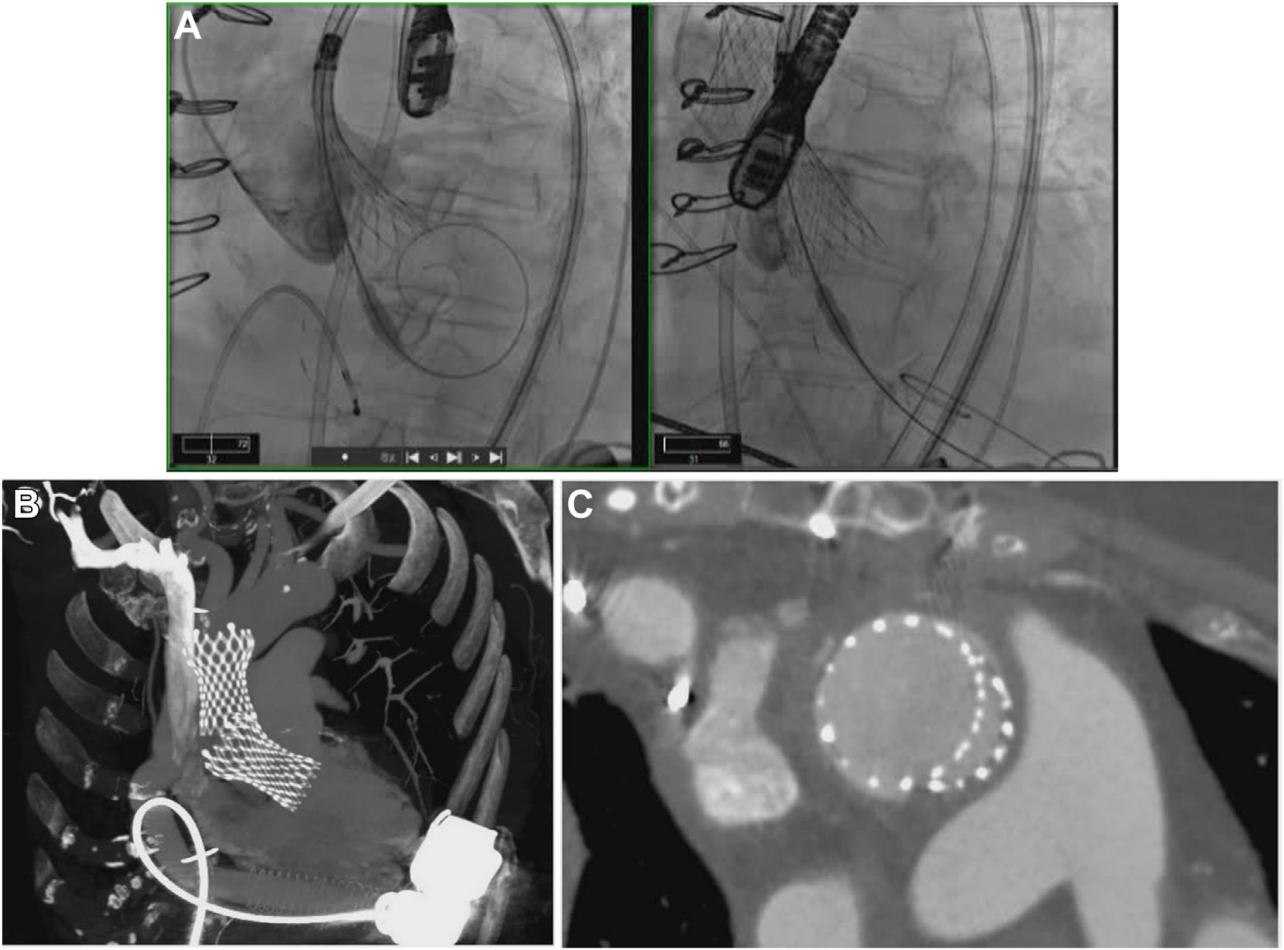
**Patient 2: (A) Second Medtronic Evolut PRO+ deployed 4.0 to 5.0 mm below the annular plane.** (**B**) Postprocedural computed tomography showing 2 stabilized Evolut valves. (**C**) Computed tomography short-axis view.

Preprocedural CTA revealed an annular perimeter of 72.1 mm, an area of 400.3 mm^2^, left ventricular outflow tract perimeter of 80.3 mm, and an annulus eccentricity index of 0.24. There was no aortic valve calcification (Figure 4). The inflow of the valve was initially deployed; however, before release, the THV slid ventricularly and landed approximately 10.0 mm below the noncoronary cusp. Transthoracic echocardiogram showed moderate PVL and mild AR. The decision was made to postdilate with a 22.0- × 60.0-mm balloon catheter. Interrogation of the valve with echocardiography revealed improvement in PVL and resolution of AR. However, it was felt that the valve remained too ventricular. A 30.0-mm gooseneck snare over a 6F AL1 guide catheter was then inserted into the right femoral artery sheath, and attempts were made to reposition the valve by initially snaring the greater curvature valve tab. This was unsuccessful, so an attempt was made by snaring the lesser curvature valve tab. This maneuver caused the valve to embolize into the ascending aorta. While maintaining the embolized valve in the ascending aorta with the snare, a pigtail catheter and J-wire were passed through the embolized valve into the left ventricle. A second Medtronic Evolut PRO+ valve and delivery system were then advanced around the aortic arch and through the embolized THV. The second THV was deployed 4.0 to 5.0 mm below the annular plane (Figure 5A, B). The valve was left in situ for 10 minutes before final release. No residual PVL was observed. The first valve was implanted along the inner curvature and migrated deep into the left ventricular outflow tract after release. The second valve was implanted at a similar depth on the noncoronary cusp and along the inner curvature but fortunately anchored onto the inflow of the first valve at the level of the aorta (Figure 5C). Retrospective CTA analysis revealed appropriate oversizing at the annulus (26%) but significant flaring of the left ventricular outflow tract dimensions, as well as a horizontal aorta with an angle of 60°.

### Outcomes and follow-up

All patients were discharged alive with less than mild AR. Six of the 7 patients were alive at 30 days postprocedure. Valve function and left ventricular function parameters at discharge and 30 days are shown in Tables 4 and 5, respectively. Systemic anticoagulation with warfarin in combination with a single antiplatelet agent (either aspirin or clopidogrel) was used following the procedure. However, 1 patient discontinued clopidogrel due to a retroperitoneal hematoma, and 3 patients discontinued aspirin in the setting of gastrointestinal bleeding. At 30 days, all patients had less than trace or trivial AR and no residual PVL. One patient was hospitalized for acute heart failure within 6 months after the procedure. One additional patient died within 60 days after the procedure; the patient presented with continued functional decline after the procedure with ongoing heart failure symptoms. A strategy of hospice or comfort measures was pursued.

**Table 5:** Pre-TAVR and 30-day echocardiogram measures.

	Patient						
	1	2	3	4	5	6	7
Pre-TAVR echocardiogram							
Left ventricular end-systolic diameter, cm	—	5.76	6.1	—	4.1	6	3.4
Left ventricular end-diastolic diameter, cm	7.3	6.3	6.6	7.1	5.3	6.3	4
30-d echocardiogram							
Left ventricular ejection fraction, %	13	33	13	17	17	13	—
Left ventricular end-systolic diameter, cm	—	3.8	—	6.8	5.6	6.2	—
Left ventricular end-systolic diameter/BSA	—	2.5	—	3.7	2.8	2.8	—
Fractional shortening, %	—	13	—	3	6	9	—
Inter ventricular septum, cm	—	0.95	—	1.07	0.6	1.1	—
Left ventricular end-diastolic diameter, cm	7	4.4	7.6	6.9	5.9	6.8	—
Left ventricular end-diastolic diameter, cm/BSA	3.7	2.6	3.3	3.7	2.9	3.2	—
Mitral regurgitation severity	Trace/trivial	Trace/trivial	Mild	Trace/trivial	None	Moderate-severe	—
Tricuspid regurgitation severity	Trace/trivial	Trace/trivial	None	Mild	Mild	Moderate	—
Aortic regurgitation severity	Trace/trivial	None	None	None	None	None	—
Paravalvular leak	None	None	None	None	None	None	—
Change in measurement from pre-TAVR to 30-d							
Left ventricular end-diastolic diameter, cm	—	−1.4	1.5	—	1.8	0.8	−3.4
Left ventricular end-diastolic diameter, cm	−0.3	−1.9	1	−0.2	0.6	0.5	—

BSA, body surface area; TAVR, transcatheter aortic valve replacement.

## Discussion

We conducted a single-center, 10-year analysis of patients undergoing TAVR to address AR in patients with CF LVAD. Our goal was to provide the institutional perspective on the frequency, treatment description, mitigation of complications and clinical outcomes of TAVR in this challenging population. There are important findings of this study (Central Illustration). First, owing to the challenges of THV anchoring, procedural planning with CTA and adequate patient selection are of utmost importance in this population. In this study, 2 patients treated with self-expanding platforms developed valve embolization or migration; however, mitigation of intraprocedural complications is important as it can lead to AR improvement and acceptable clinical outcomes. Finally, despite the increased risk, elimination or reduction in AR can be safely achieved in carefully selected patients.

**Central Illustration:**
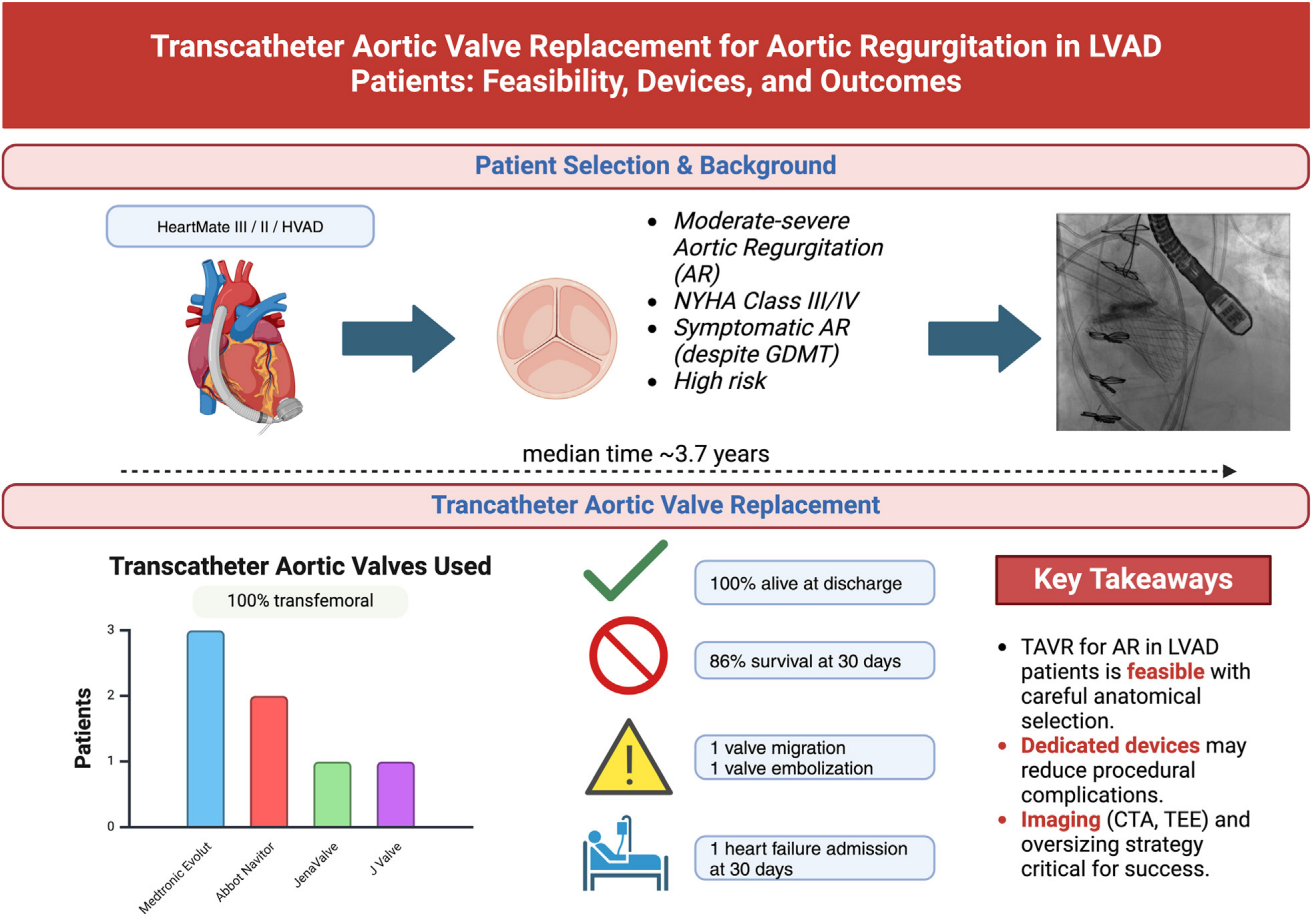
**TAVRfor AR in LVAD patients: feasibility, devices, and outcomes.** CTA, computed tomography angiography; LVAD, left ventricular assist device; TAVR, transcatheter aortic valve replacement; TEE, transesophageal echocardiography.

Aortic regurgitation is a commonly encountered valvular heart disease in patients with CF LVAD, where it is estimated that half of patients will develop moderate to severe AR 2 years after LVAD implantation. Worsening of AR can lead to the progression of left-sided and right-sided heart failure, decreasing forward LVAD flow and consequently impairing organ perfusion.^22^ When conservative management of significant and symptomatic AR fails, surgical intervention may be considered, although the risks associated with a reoperation are significant.^23^ Current clinical practice guidelines assign a class III (harm) indication for current commercially available THV, only in patients at prohibitive surgical risk and in whom valve calcification and annular size are favorable, TAVR can be attempted.^24^

To date, there are no consensus or guideline recommendations about the use of TAVR to treat AR in patients with CF LVAD, where most literature available describes the use of off-label TAVR in high-risk, inoperable patients.^25^ Similar to native AR in patients without LVAD, anatomic challenges are often encountered when using commercially available THV dedicated to treating aortic stenosis. On CTA, the aortic annulus is often larger and noncalcified, which makes anchoring of THV challenging and fraught with the potential complications of valve migration, embolization, a second THV, surgical conversion, and significant residual PVL, all of which lead to lower procedural success and increased mortality.^26^ A certain degree of THV oversizing is recommended to avoid such complications, but this runs the risk of complications such as permanent pacemaker implantation or aortic root rupture.^27^, ^28^, ^29^

In addition, the LVAD flow toward the left ventricle increases the risk of THV migration.^22^ Owing to its rarity, the treatment description of acute valve migration during TAVR has been limited to observational studies.^17^, ^18^, ^19^ Chawla et al^30^ describe a framework and algorithm to help operators address this rare but significant complication. Balloons, snares, and/or bioptomes can be used to secure and/or reposition the embolized valve into a suitable position free from branch vessels or other structures.^30^

Two of our cases were complicated by THV migration, and both these cases used self-expandable THV; one was treated successfully by snaring and repositioning the migrated valve, and the second required a second THV after aortic embolization. It has been shown that self-expandable THV has a higher, but not statistically significant, risk of valve migration or embolization.^31^ The “Anatomical classification and dual anchoring theory to Optimize the TAVR strategy for pure severe Aortic regurgitation” aims to create an anatomical classification to evaluate the safety and efficacy of transfemoral TAVR using self-expanding platforms for patients with severe pure AR.^32^ Based on annulus, left ventricular outflow tract, and ascending aorta CTA characteristics, patients can be classified into 4 categories and predict TAVR feasibility. The first patient that presented valve migration had suboptimal image quality, which limited accuracy of the annular measurements. Independent retrospective analysis of the CTA showed that the oversize at the annulus was insufficient at 10.5 %, with a limited oversize at the ascending aorta (12.1%) and no oversize at the level of the left ventricular outflow tract with an average diameter of 33.4 mm. The second patient had an appropriate oversizing at the annulus (26%) but significant flaring of the left ventricular outflow tract dimensions as well as a horizontal aorta with an angle of 60°. Inappropriate oversizing to ensure adequate valve fixation at 2 levels might have led to the embolization of the device. In addition, poor quality imaging study and horizontal angulation of the ascending aorta were risk factors associated with these complications. Key factors influencing procedural success with TAVR devices include careful analysis of preprocedural imaging. Accurate measurements of the aortic annulus, left ventricular outflow tract, and ascending aorta were crucial for procedural planning. In addition to CTA, the use of multimodality imaging—including transthoracic and transesophageal echocardiography—was emphasized to assess valve morphology, left ventricular size and function, severity of AR, and hemodynamics. Procedural planning allowed us to determine the adequate oversizing of the THV relative to the annular dimensions. Our experience with TAVR in the setting of LVAD-related AR is that annular oversizing of at least 15% to 20% is necessary to reduce the risk of valve embolization and migration. Achieving secure anchoring at both the annular and subannular levels was emphasized, especially given the lack of calcification in native AR cases, which makes anchoring inherently more difficult. The geometry of the aortic root and ascending aorta also played an important role. Patients with horizontal aortas or significant dilation of the ascending aorta posed additional challenges, requiring careful consideration of device selection and deployment strategy.

In our study, all patients were discharged alive; however, 2 patients died within 3 months after the procedure, and 1 was readmitted for acute heart failure. In a study of 87 patients who underwent TAVR, cardiogenic shock events, bleeding, and vascular complications were higher in patients who underwent surgical valve replacement (61 patients), with no statistical differences in 30-day readmission and mortality.^33^ In addition, in a meta-analysis of 242 patients receiving TAVR vs 116 patients undergoing surgical replacement, acute kidney injury, and costs were significantly higher in the SAVR group, with no significant difference in median overall survival (18 months).^34^ Nevertheless, quality of life and functional status improve after TAVR in patients with LVAD.^17^

Finally, 2 cases were successfully performed using investigational THV dedicated to address native valve AR: J-Valve (JC Medical)^35^ and JenaValve Trilogy.^36^ These THV rely on anchoring rings (J-Valve) and locators (JenaValve Trilogy) for leaflet fixation. Therefore, they can provide effective anchoring in noncalcified anatomies.^16^^,^^26^^,^^37^ Both valves are undergoing prospective clinical evaluation in the United States, including a dedicated registry for patients with LVAD (NCT06455787 and NCT06608823).

### Limitations

The findings from this single-center study should be considered in the context of several important limitations. First, given the retrospective nature of our observations, single-center design, and small sample size, our findings should be considered hypothesis-generating rather than confirmatory; our goal was to describe our institutional experience with TAVR in this subset of patients. Finally, long-term follow-up data are missing. Thus, we are describing outcomes at discharge and at 30 days.

## Conclusions

In summary, TAVR is feasible in carefully selected patients with native AR associated with a LVAD. Mitigation of procedural complications may be necessary when using commercially available devices. Ongoing studies with dedicated devices will inform risks and benefits of TAVR in this challenging population.
